# Management of Refractory Noninsulinoma Pancreatogenous Hypoglycemia Syndrome with Gastric Bypass Reversal: A Case Report and Review of the Literature

**DOI:** 10.1155/2015/384526

**Published:** 2015-10-07

**Authors:** Bhavana B. Rao, Benjamin Click, George Eid, Ronald A. Codario

**Affiliations:** ^1^Department of Internal Medicine, University of Pittsburgh Medical Center, Pittsburgh, PA 15213, USA; ^2^Division of Bariatric Surgery, Allegheny Health Network, Pittsburgh, PA 15212, USA; ^3^Division of Endocrinology, Department of Internal Medicine, VA Pittsburgh Healthcare System, Pittsburgh, PA 15240, USA

## Abstract

*Background*. Roux-en-Y gastric bypass (RYGB) is a commonly performed, effective bariatric procedure; however, rarely, complications such as postprandial hypoglycemia due to noninsulinoma pancreatogenous hypoglycemia syndrome (NIPHS) may ensue. Management of refractory NIPHS is challenging. We report a case that was successfully treated with RYGB reversal. *Case Report*. A 58-year-old male with history of RYGB nine months earlier for morbid obesity presented for evaluation of postprandial, hypoglycemic seizures. Testing for insulin level, insulin antibodies, oral hypoglycemic agents, pituitary axis hormone levels, and cortisol stimulation was unrevealing. Computed tomography (CT) scan of the abdomen was unremarkable. A 72-hour fast was completed without hypoglycemia. Mixed meal testing demonstrated endogenous hyperinsulinemic hypoglycemia (EHH) and selective arterial calcium stimulation testing (SACST) was positive. Strict dietary modifications, maximal medical therapy, gastrostomy tube feeding, and stomal reduction failed to alleviate symptoms. Ultimately, he underwent laparoscopic reversal of RYGB. Now, 9 months after reversal, he has markedly reduced hypoglycemia burden. *Discussion*. Hyperfunctioning islets secondary to exaggerated incretin response and altered intestinal nutrient delivery are hypothesized to be causative in NIPHS. For refractory cases, there is increasing skepticism about the safety and efficacy of pancreatic resection. RYGB reversal may be successful.

## 1. Introduction

Roux-en-Y gastric bypass (RYGB) is frequently performed for the management of morbid obesity. While it offers significant and sustained weight reduction and favorably impacts several metabolic parameters, we are now encountering some unique and challenging postprocedure complications. One such long-term complication is postprandial hypoglycemia. A rare cause of postprandial hypoglycemia is noninsulinoma pancreatogenous hypoglycemia syndrome (NIPHS). NIPHS is characterized by endogenous hyperinsulinemic hypoglycemia (EHH) with positive selective arterial calcium stimulation testing (SACST) and negative imaging studies for insulinoma.

When first described, gastric bypass patients with NIPHS were noted to have diffuse hyperplasia of the beta cells of the pancreatic islets, akin to nesidioblastosis, resulting in inappropriately elevated levels of insulin [1]. Subsequent studies proposed that an exaggerated incretin hormonal response to meals stimulated this hyperplasia of the islet beta cells [2].

Historically, management of mild cases has included dietary modifications such as carbohydrate-restricted diets and medications such as acarbose, verapamil, octreotide, and diazoxide [3, 4]. For patients with severe or refractory symptoms, gradient-guided partial or total resection of the pancreas has been performed; however, hypoglycemic events persist in majority of the patients [2, 3]. Gastric bypass reversal for the treatment of refractory NIPHS has been sparsely described, with equivocal results [2, 5–7]. We report a case of severe refractory NIPHS successfully managed with gastric bypass reversal.

## 2. Case Report

A 58-year-old Caucasian male presented to our institution after suffering a generalized tonic-clonic seizure lasting five minutes, followed by a brief postictal state with complete amnesia to the event. A home glucometer read less than 70 mg/dL, so he was transported to our facility for further evaluation. His past medical history included coronary artery disease necessitating coronary artery bypass surgery, ischemic cardiomyopathy with biventricular intracardiac defibrillator placement, hypertension, type 2 diabetes, and RYGB 9 months earlier for morbid obesity. The patient had been a diabetic for 4 years prior to the RYGB and had been maintained on a relatively stable regimen of subcutaneous insulin with metformin, with hemoglobin A1c ranging between 8.5 and 10%. Following the procedure, he had continued to be on the same regimen with no reported side effects. Upon admission, he was hemodynamically stable and afebrile, with unremarkable physical examination and no focal neurological deficits. Complete cell count and metabolic panel were within normal limits except for blood glucose (BG) level of 69 mg/dL, notably after 25 gm of dextrose was administered intravenously, en route to the hospital. Electroencephalogram and computed tomography (CT) scan of his head were nonrevealing. Of note, in the month prior to RYGB, his weight was 287 pounds with body mass index (BMI) of 42 kg/m^2^ and hemoglobin A1c (HbA1c) of 9.6%. At the time of admission, he reported a 75-pound weight loss, with decrease in BMI to 30 kg/m^2^ and HbA1c to 5.5%. His insulin and oral antihypoglycemic regimen were discontinued after this episode of presumed hypoglycemic seizure.

Over the next 6 months, the patient had several more hypoglycemic seizures that occurred several hours after eating, with perievent BG ranging from 30 to 80 mg/dL. CT scan of the abdomen was notable for surgical changes and a 2.1 cm adrenal nodule. Laboratory testing revealed normal levels of urine and plasma metanephrines, random serum and 24-hour urine cortisol, and appropriate response to cortisol stimulation and low dose dexamethasone suppression test. Testing for insulin antibodies and oral hypoglycemics was negative and a 72-hour fast was completed without hypoglycemia. The patient was advised to eat small meals every 4 hours, rich in proteins and complex rather than simple carbohydrates, and educated on continuous blood glucose monitoring as well as the use of a glucagon kit. However, the hypoglycemic seizures persisted.

Upon further testing, induced hypoglycemia with 75% carbohydrate meal intake showed BG of 36 mg/dL three hours later, with inappropriately elevated C-peptide (7.8 ng/mL; normal [nr] <0.2 in hypoglycemia), proinsulin (11.5 pmol/L; nr: <5 pmol/L in hypoglycemia), and insulin levels (22.4 *μ*IU/mL; nr < 3 in hypoglycemia), consistent with EHH. He was sequentially trialed on maximum doses of acarbose (50 mg TID), octreotide (100 mcg TID), and diazoxide (50 mg BID) without resolution of the hypoglycemic episodes. Subsequent SACST revealed a 2-, 3-, and 9-fold increase in insulin levels, 120 seconds after injection of calcium gluconate, in the gastroduodenal, midsplenic, and proper hepatic arteries, respectively, suggestive of hyperfunctioning islets or islet hypertrophy localized to the head or body of the pancreas.

Percutaneous gastrostomy tube placement to the remnant stomach with intermittent tube feeding was attempted; however, with one missed feeding, he again suffered a hypoglycemic seizure. Endoscopic gastrojejunal stomal reduction to delay transition of food was performed. Symptoms improved temporarily but with ultimate recurrence of hypoglycemic episodes. The patient had over 25 hospitalizations for hypoglycemia during a period of 2 years. In view of the life-threatening nature of his episodes, the patient underwent laparoscopic reversal of RYGB three years after original procedure, with resection of the roux limb and restoration of normal anatomy by creation of gastrogastrostomy. Currently, the patient is nine months after reversal, weighs 230 pounds with a BMI of 34 kg/m^2^ and HbA1c of 5.5, and has had only episode of hypoglycemic seizure since the surgery. This occurred eight months after reversal when he was working outdoors after missing a meal. His blood glucose dropped to 50 mg/dL but he was unable to hear the alarm of the continuous glucose monitor due to a noisy environment. With the exception of this singular event, the patient has not had any other episodes of hypoglycemia or hospitalizations.

## 3. Discussion

Over the last decade, several studies have contributed to our understanding of the underlying pathologic mechanisms in NIPHS and influenced management strategies.

The most widely accepted hypothesis proposes increased levels of the incretin hormone glucagon-like peptide-1 (GLP-1) leading to islet cell hyperplasia, postprandial hyperinsulinemia, and subsequent hypoglycemia [8]. Based on this, recent trials have evaluated GLP-1 analogs [9] and GLP-1 receptor antagonists [4] for treatment of postprandial hypoglycemia with promising preliminary results. However, islet cell expansion is not consistently observed in pathological specimens of patients managed with pancreatectomy [1, 2]. Also, a retrospective assessment of 15 patients who had undergone pancreatic resection reported persistent hypoglycemic symptoms in 77% of patients after the procedure [3].

Refinement of the previously mentioned hypothesis proposes that the insulin dysregulation after RYGB is a result of rapid delivery of nutrients to the distal intestine, leading to an exaggerated response of the incretins GLP-1 and gastric inhibitory peptide (GIP), which causes postprandial hyperinsulinemia/hypoglycemia without necessarily leading to islet cell hyperplasia [10]. This was first suggested by McLaughlin et al. in 2010 when they noted that, by inserting a gastrostomy tube and directly feeding the remnant stomach, the hypoglycemic episodes were prevented, thus implicating altered nutrient delivery as a provocative factor [10]. This theory was further supported by an observation that, with laparoscopic reversal of gastric bypass to normal anatomy or with revision of bypass by performing modified sleeve gastrectomy, decrease in the frequency of hypoglycemic episodes and resolution of neuroglycopenia occurred in 4 patients with refractory post-RYGB NIPHS [6]. Notably, each of these patients had been successfully trialed with a gastrostomy tube to the remnant stomach prior to the reversal. Himpens et al. also reported success with bypass reversal in 1 patient [5].

Failure of resolution of symptoms with gastric bypass reversal was first described by Patti et al. in one patient whose symptoms persisted despite maximal medical therapy, takedown of bypass, and 80% distal pancreatectomy, and eventually Whipple's procedure was performed [2]. Furthermore, Lee et al. conducted mixed meal challenges in two patients who had undergone reversal of RYGB for NIPHS and noted no improvement in the hyperinsulinemic hypoglycemia, despite improvement in GLP-1 levels. Interestingly, they detected an increase in GIP levels after reversal, not observed in previous studies, which may have contributed to reversal failure [7].

In our patient, RYGB was followed by significant weight reduction and remission of diabetes but was complicated by development of NIPHS nine months postoperatively, refractory to dietary modification and medical therapy. In a prior case series, time to development of this complication ranged from 1 to 56 months and the cause for this variability is as yet unclear [3]. Gastrostomy tube feeding to the remnant stomach reduced the number of hypoglycemic episodes, but, without strict adherence, his symptoms recurred, which suggests possible alternate mechanisms which do not rely on food intake stimulated pathways and may merit further investigation. Use of continuous glucose monitor enabled him to identify and correct episodes of hypoglycemia and thus prevent seizures; however, multiple breakthrough seizures occurred. Endoscopic stomal reduction, with the objective of prolonging gastric emptying time, was successful for a short period of time; however, hypoglycemic episodes persisted. Surgical procedures such as gastric pouch reduction or adjustable band placement have been trialed; however, limited data exists on the mechanism and benefit of these measures. In our particular case, the roux limb was resected due to an intraoperative finding of severe adhesions and possible small bowel anastomosis in two different segments of the roux limb, which may have further hastened nutrient delivery to his distal intestine and thus exacerbated the hypoglycemia. Following bypass reversal, he has not required hospitalization and his overall burden of hypoglycemia has been dramatically reduced.

## 4. Conclusion

Our understanding of post-RYGB NIPHS, its etiology, and appropriate surgical procedures for its management is limited by the fact that this syndrome is rare and only recently recognized. There is now increasing skepticism about the rationale, effectiveness, and safety of pancreatic resection for this condition. Critical appraisal of the literature reveals few prior reports of gastric bypass reversal, each with variable results, which may be attributable to differences in underlying anatomy and variations in surgical techniques. Here, we report the successful treatment of a case of refractory NIPHS with reversal of RYGB. Further investigations to better elucidate the hormonal milieu before and after reversal are necessary to facilitate better management of this challenging condition.
